# Risk factors of myasthenia crisis after thymectomy among myasthenia gravis patients: A meta-analysis: Erratum

**DOI:** 10.1097/MD.0000000000029480

**Published:** 2022-06-03

In the article, “Risk factors of myasthenia crisis after thymectomy among myasthenia gravis patients: A meta-analysis”, which appears in Volume 99, Issue 1 of *Medicine*, the n value for “Records after duplicates removed” appeared incorrectly in Figure 1 when originally published. This number has been corrected from 130 to 407.
